# Adult Vaccination in the United Arab Emirates—A Physicians' Knowledge and Knowledge Sources Study

**DOI:** 10.3389/fpubh.2022.865759

**Published:** 2022-04-14

**Authors:** Hiba J. Barqawi, Kamel A. Samara, Mahmoud S. Hassan, Firas B. Amawi

**Affiliations:** ^1^Department of Clinical Sciences, College of Medicine, Sharjah, United Arab Emirates; ^2^College of Medicine, University of Sharjah, Sharjah, United Arab Emirates; ^3^University Hospitals Coventry and Warwickshire NHS Trust, Conventry, United Kingdom; ^4^Dr. Sulaiman Al Habib Hospital, Dubai, United Arab Emirates

**Keywords:** adult vaccination, United Arab Emirates (UAE), physician knowledge, adult immunization, vaccine preventable disease

## Abstract

**Background:**

A lack of knowledge on adult vaccination has been documented among physicians. They play a critical role in promoting adult vaccines. This study aimed to review the status of adult vaccination in the United Arab Emirates (UAE) and evaluate physicians' knowledge and knowledge sources regarding adult vaccines.

**Methods:**

Local, regional, and global adult vaccination guidelines were reviewed. A 40-item questionnaire was used to collect data from physicians from June to October 2020, using convenience and snowball sampling. Knowledge score was calculated, and predictors identified using Mann–Whitney *U* and Kruskal–Wallis *H*-tests. Ordinary Least Squares regression was used for Multivariate Analysis.

**Results:**

A total of 500 responses were included. A quarter were internists, and another quarter were family physicians. Fifty-seven percent were medical interns and residents. Both perceived and actual knowledge of adult vaccination were low. Bivariate analysis showed knowledge depending on department, level of training, workplace, and perceived knowledge. All remained significant after multivariable regression except workplace. International and local guidelines were the most common knowledge sources. Forty-two percent were unable to access the local guidelines.

**Conclusions:**

Physicians' knowledge was poor and local guidelines were not clear or easily accessible. Participants were highly receptive to guidance and practice with adult vaccines.

## Background

The World Health Organization's (WHO) third strategic objective aims to ensure that “the benefits of immunization are equitably extended to all people.” To ensure this, immunization must be provided to all children, adolescents, and adults. In fact, life-course immunization is among the recommended actions for three of the six strategic objectives for attaining the WHO's Global Vaccine Action Plan 2011–2020 (1). However, there are many recognized barriers to adult vaccination, the most prominent and encapsulating being vaccine hesitancy. It is defined as the delay or refusal of vaccines despite availability and serves as a continuum between proponents and opponents of vaccination. Other barriers include a lack of public support for adult vaccination, complexity of adult vaccination schedules, and prioritization of infant vaccination programs (2).

Globally, the burden of vaccine preventable diseases (VPDs) and vaccine uptake rates have not been explored thoroughly in adults. In the United States of America (USA), VPDs in adults cost the healthcare system $9 billion every year (3). This number will only grow as the United Nations projects that between 2015 and 2030, the number of people in the world aged 60 years or older will grow by 56%, reaching a total of 1.4 billion people (4). Hence, many global organizations have called for increased focus on adult immunizations, particularly in the vulnerable aging population.

Healthcare providers (HCPs) play a critical role in fostering vaccine acceptance among those that are hesitant (5). Out of the six themes that influence the willingness to get immunized, five, including attitudes, beliefs, risk perception and health practices, involved physicians (6). Yet, HCPs have low awareness and leadership when it comes to adult vaccination, possibly due to a lack of training (2). In fact, a lack of knowledge, initiative, belief or pro-vaccination practices has been documented among physicians (5, 7–12).

Hence, there has been a newfound interest in evaluating physicians' knowledge regarding adult vaccines. Yet very few studies have looked at this in the Middle East and North Africa (MENA) region and even less locally. In the United Arab Emirates (UAE), vaccination has been an under-researched topic with only a few studies exploring specific vaccines, such as influenza or Human Papilloma Virus (HPV) (13–15). However, no studies have looked at physicians and their role in promoting general adult vaccination. The aims of this study were to (a) perform a desk review of the status of adult vaccination in the UAE and (b) undertake original research to evaluate physicians' knowledge and knowledge sources regarding adult vaccines.

## Methods

### Adult Vaccination Guidelines Review

For the first aim of this study, a desk review of adult vaccination was performed by reviewing the guidelines for three countries, USA, Saudi Arabia (KSA), and UAE. For USA, the Centers for Disease Control and Prevention (CDC) guidelines were used (16). For KSA, the schedule published by the Ministry of Health was used (17). In the UAE, there are three main health authorities: the Dubai Health Authority (DHA), responsible for the Emirate of Dubai (3.4 million; 34.7% of the population), the Abu Dhabi Department of Health (DOH/HAAD/SEHA), responsible for the Emirate of Abu Dhabi (2.9 million; 29.6% of the population), and finally, the Ministry of Health and Prevention (MOHAP) focusing on the remaining Emirates (3.5 million; 35.7% of the population) (18–20). Out of the three health authorities, only the DHA had a full adult vaccination schedule (21). The DOH's and MOHAP's schedules did not touch upon routine adult immunization; they focused on the vaccines for high-risk groups (22). Given the aims of the study, the DHA's schedule was adopted as the prototype for the UAE.

Both the global and local guidelines regarding adult vaccination were reviewed, compiled, compared, and contrasted to highlight areas of deficit. Additionally, the guidelines were simplified and presented as a short schedule to help physicians quickly determine a patient's need for a vaccine.

### Study Population

Thereafter, a cross-sectional, descriptive study was designed to collect original data from UAE physicians all over the country. It was conducted between the months of June and October 2020 using convenience and snowball sampling. Participants were approached through email, phone, WhatsApp, and other social media networks making use of contact details listed on the health authority websites. Any physician with at least 1 year of experience who is currently practicing in the UAE was eligible to participate. The minimum sample size needed was 385, assuming an expected prevalence of 50%, a margin of error of 5%, and 95% confidence. The number was increased by 20% to 460 to account for non-response. In total, 534 questionnaires were collected out of which 34 were excluded due to them not fulfilling the inclusion criteria.

### Questionnaire Development

A 40-item questionnaire was developed after reviewing the adult vaccination literature and guidelines. Due to the COVID-19 pandemic, data was collected online. Google Forms was used, ensuring the user's privacy by not collecting any personal identifiers. The self-administered questionnaire consisted of two main sections: demographics and adult vaccination knowledge. It included 5-item Likert scales, true and false questions, as well as multiple-choice questions. The questions explored physicians' (a) perceived knowledge, (b) the vaccines they would recommend at different age groups, and (c) their knowledge sources and experience with local guidelines.

The questionnaire was pilot tested several times on different physicians; all provided feedback was evaluated and incorporated, if appropriate. This study was reviewed and approved by the Research Ethics Committee at the University of Sharjah (Reference Number: REC-20-04-09-01-S). It was conducted in accordance with all relevant guidelines and regulations. Informed consent was obtained from all participants.

### Data Analysis

The data was exported from Google Forms and imported into Python 3.9 for analysis. Data cleaning and pre-processing was performed. A knowledge score was calculated by categorizing the adult vaccines into four main groups:

- Those that are always recommended, regardless of risk factors, previous exposure, or vaccination status.
- Those that are recommended unless previous exposure or vaccination is documented.
- Those that are recommended based on the patients' risk factors only.
- Those that are not routinely recommended.

For every vaccine that a participant recommended, 2 points were awarded if the vaccine is recommended for all healthy adults, or 1 point awarded if it is recommended for all healthy adults lacking immunity, or 1 point deducted if the vaccine is only indicated for specific risk factors or not routinely indicated. All the points were added to calculate the knowledge score. The maximum possible score was 31 while the minimum was −11.

For univariate analysis, the normality of the knowledge score was evaluated using both Q-Q plots and a Shapiro-Wilk test. All reported percentages were calculated by excluding the missing values. All demographic variables and perceived knowledge were evaluated as predictors for the knowledge score. Bivariate analyses were conducted to identify significant predictors using Mann–Whitney *U* and Kruskal–Wallis *H*-tests, the former for binary variables and the latter for those with more than two categories. The cut-off for significance was a *P* < 0.05.

All determinants were categorical and hence dummy coded except for perceived knowledge. Ordinary Least Squares regression was used. Heteroskedasticity was tested for using a Studentized Breusch-Pagan test. No outliers were detected. For the linear regression model, the minimum number of cases was met, which was calculated using 50 + 8*m*, where *m* is the number of predictors. No interactions were explored. F score and R-squared values were calculated for the model. All *P*-values reported are two-sided and all confidence intervals are profile confidence intervals.

## Results

### Adult Vaccination Guidelines

Adult vaccines can be divided into two main groups: those recommended for the general adult population and those indicated for individuals with specific risk factors such as chronic lung or heart disease, diabetes, compromised immune system, travel, or high-risk occupations (16). Table 1 shows the guidelines for the UAE, KSA, and USA. UAE guidelines use three age groups: 18–59, 60–64, and 65 or older. The KSA and CDC guidelines take a more granular approach and utilize five age groups: 18–26, 27–49, 50–59, 60–64, and 65 or older.

**Table 1:** USA, KSA, and UAE adult vaccination schedules.

**Vaccine**	**CDC**	**KSA**	**UAE (DHA)**
Influenza I/R	Everyone	Everyone	Everyone
Influenza Live	<50 years	No info	Not available
Tdap	Everyone	Everyone	Everyone
MMR	NEI/PD	NEI/PD	NEI/PD
Varicella	NEI/PD	NEI/PD	NEI/PD
Zoste Recomb.^*^	≥50 years	≥50 years	Not indicated
Zoster Live^*^	≥60 years	≥50 years	≥60 years
HPV	19–26: routinely; 27–45: SCD	16–25:catch up	19–59: optional
PPSV	OIRF; ≥65 years routinely	≥65 years	≥65 years
PCV13	OIRF; ≥65 needs SCD	≥65 years	OIRF
Hepatitis B	OIRF	OIRF	OIRF
MCV4^**^	OIRF	OIRF	OIRF
MenB^**^	16–23 years based on SCD	No info	OIRF
Hepatitis A	OIRF	OIRF	OIRF
Hib	OIRF	OIRF	OIRF

* *For Zoster, the KSA and UAE guidelines do not distinguish between the two types of vaccines*.

** *For meningococcal vaccines, UAE adult vaccination guidelines did not distinguish between both types*.

*For contraindications and dosing, please refer to original guidelines, links of which can be found in the references section. Influenza I/R, influenza inactivated/recombinant; Zoster recomb, zoster recombinant; NEI/PD, no evidence of immunity or previous disease; OIRF, other indication or risk factor*.

The three schedules' recommendations are aligned for influenza, Tetanus toxoid, reduced diphtheria toxoid, and acellular pertussis (Tdap), Measles, Mumps, and Rubella (MMR), and varicella vaccines. For the pneumococcal vaccine, the CDC and UAE recommend Pneumococcal polysaccharide vaccine 23 (PPSV23) routinely while KSA recommends both PPSV23 and Pneumococcal conjugate vaccine (PCV13). As for the hepatitis B vaccine, the KSA guidelines routinely recommend it whereas the UAE and the CDC do not. Finally, for hepatitis A and Haemophilus influenzae type B (Hib), they were not discussed in the KSA guidelines and the UAE guidelines only indicated them for those with other risk factors.

Considering the unique healthcare system, demographic distribution and public health policies of the UAE, an outline of the recommended adult vaccines is presented in Table 2. It is adopted from the CDC's schedule, considering the regional and local meningococcal and hepatitis B guidelines. With regards to influenza, only the inactivated/recombinant vaccine was included for two reasons (a) the UAE's guidelines state that the live vaccine is not available (21) and (b) the CDC recommends it over the live one (16). Similarly, for the zoster vaccine, the recombinant was included given the CDC's recommendation.

**Table 2:** Recommended adult vaccination schedule.

**Vaccine**	**19–26 years**	**27–49 years**	**50–59 years**	**60–64 years**	**65 years and above**
Influenza I/R	1 dose annually for adults				
Td/Tdap	1 dose of Td every 10 years with the first dose being Tdap for all adults				
MMR	1 or 2 doses if no evidence of immunity or previous disease				Not recommended
Varicella	2 doses if no evidence of immunity				
Zoster Recomb.	Not recommended		2 doses regardless of previous exposure		
HPV	2 or 3 doses	2 or 3 doses; based on SCD	Not recommended (age 45 and above)		
PPSV23^*^	1 or 2 doses if other indications or risk factors				1 dose for all adults
PCV-13	1 dose if other indications or risk factors				1 dose based on SCD
Hepatitis B	3 doses if no evidence of immunity				
MCV4	1 or 2 doses if other indications or risk factors				
Hepatitis A	2 or 3 doses if other indications or risk factors				
Hib	1 or 3 doses if other indications or risk factors				

* *The total number of PPSV23 doses should be two; hence, it is not required to give a dose after the age of 65 if a patient has already received two. Additionally, the second dose should be at least 5 years from the last dose*.

*The table is color-coded according to five levels of recommendation:*

• Routinely indicated for all healthy adults.

• Recommended if immunity is not shown.

• Recommended if there are other risk factors or indications.

• Requires shared clinical decision making.

• Not recommended.

*Evidence of immunity entails either a written documentation of vaccine administration or laboratory evidence of immunity or disease. SCD (Shared Clinical Decision) recommendations are not for everyone but based on a discussion between the healthcare provider and the patient. Note that some vaccines have contraindications, such as an egg allergy with the influenza vaccine. Refer to CDC guidelines for an exhaustive list of contraindications and dosing instructions. Influenza I/R, influenza inactivated/recombinant; zoster recomb, zoster recombinant*.

Additional points and comments can be added to the schedule to make it more exhaustive; however, the goal was to present a simple yet accurate representation of the possible vaccinations at different age groups, serving as a quick reference sheet. While indications and risk factors are not highlighted, situations where a physician needs to probe further are indicated. However, still, a few comments are warranted:

- For meningococcal, it is important to vaccinate college students living in residential dorms and military recruits.
- For hepatitis A, while the guidelines recommend administering the vaccine based on indications, the importance of geographical area as an indication cannot be understated. Further studies should evaluate hepatitis A outbreaks in the country to delineate areas that require routine hepatitis A vaccination.

### Adult Vaccination Knowledge Among UAE Physicians

#### Demographics

Table 3 presents the characteristics of the participating physicians. Two hundred and ninety-eight (59.6%) physicians were female with 370 (74%) aged 35 or under. A quarter of the participating physicians were internists and another quarter were family physicians. Medical interns and residents consisted of 57% of the participants with the rest being split between general practitioners, specialists, and consultants. Seventy percent of the participants were employed at a governmental hospital. The doctors were equally split among the three health authorities in the country.

**Table 3:** Participant demographics, bivariate analyses, and average knowledge for each group.

**Feature**	***n* (%)**	**Result/Score†**	**Feature**	***n* (%)**	**Result/Score†**
**Sex (*****N*** **= 500)**		MWU; P=0.313	**Level of Training (N=500)**		KW; *P* = 0.029*
Female	298 (59.6%)	9.641	Intern house officer	134 (26.8%)	9.627
Male	202 (40.4%)	9.213	Resident	153 (30.6%)	9.353
**Age (*****N*** **= 500)**		KW; *P* = 0.222	General practitioner	82 (16.4%)	9.378
≤25 years	183 (36.6%)	9.235	Specialist	53 (10.6%)	7.953
≥26 years but ≤35 years	187 (37.4%)	9.086	Senior specialist	18 (3.6%)	7.639
≥ 36 years but ≤ 45 years	54 (10.8%)	10.843	Consultant	60 (12.0%)	11.417
≥ 46 years	76 (15.2%)	9.993	**Workplace (N=500)**		KW; *P* = 0.006*
**Citizenship (*****N*** **= 500)**		KW; *P* = 0.690	Government hospital	356 (71.2%)	9.058
Other Arab	304 (60.8%)	9.457	Private clinic/hospital	90 (18.0%)	9.939
Local (Emirati)	115 (23.0%)	9.117	Primary healthcare	54 (10.8%)	11.389
Non-Arab	81 (16.2%)	10.006	**Patients seen in a week (*****N*** **= 500)**		KW; *P* = 0.092
**Department (*****N*** **= 364)**		KW; *P* < 0.0005*	1–19	110 (22.0%)	8.573
Internal medicine	88 (24.2%)	10.023	20–49	210 (42.0%)	9.407
Family medicine	86 (23.6%)	11.424	50 and above	180 (36.0%)	10.086
Pediatrics	42 (11.5%)	9.345	**Perceived knowledge (*****N*** **= 500)**		KW; *P* < 0.0005*
Others	148 (40.7%)	7.851	Not at all	35 (7.0%)	5.129
**Health Authority (*****N*** **= 498)**		KW; *P* = 0.154	I know a few	133 (26.6%)	8.075
MOHAP	184 (36.9%)	8.916	I know the important ones	210 (42.0%)	9.817
DHA	161 (32.3%)	9.839	I know most of them	98 (19.6%)	11.724
DOH/ HAAD/ SEHA	153 (30.7%)	9.85	I know all of them	24 (4.8%)	11.25

† *For categories (such as Health Authority), this column presents the results of the bivariate test; for possible values, this column presents the average knowledge score. Note that two physicians did not specify a health authority and hence were regarded and treated as missing. All medical interns were not assigned a department. All significant P-values have asterisks after them*.

**Figure 1:**
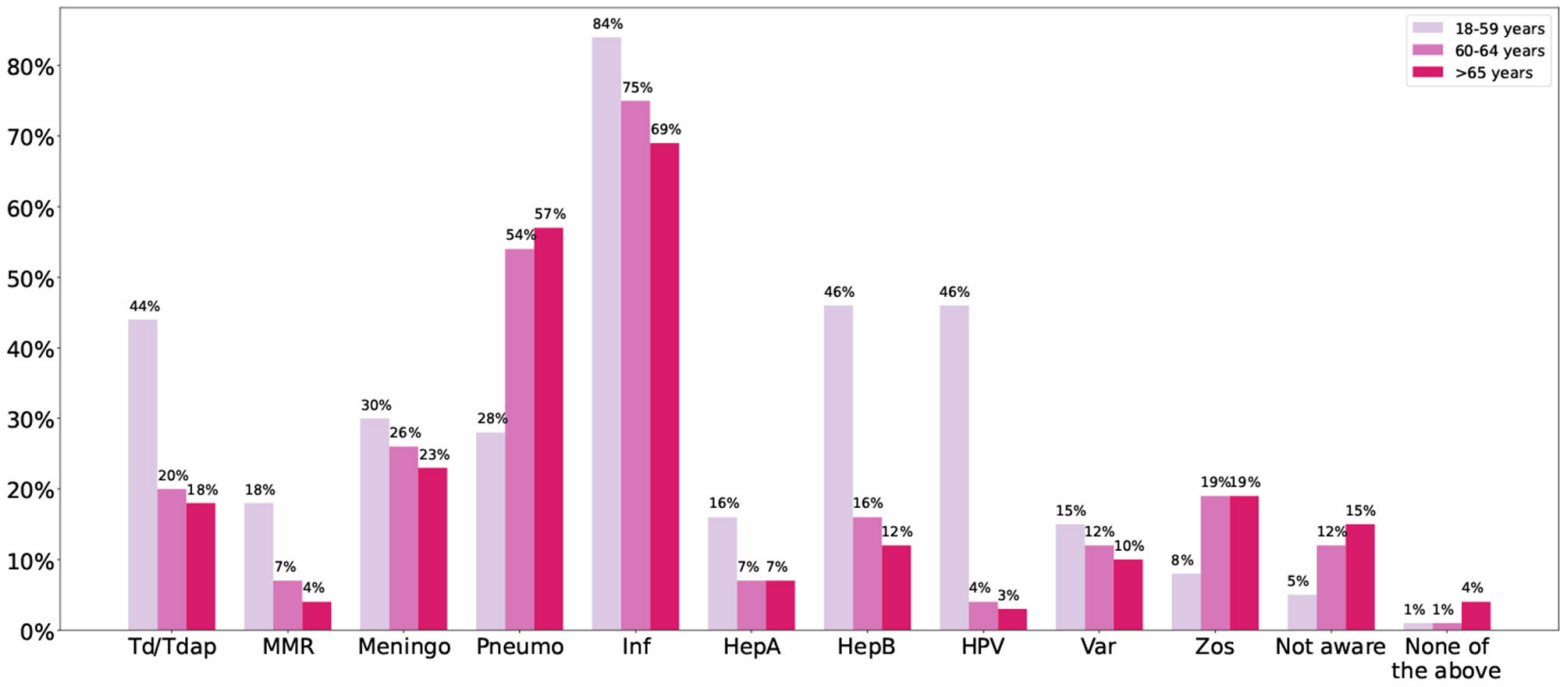
The figure shows the percentage of physicians that would recommend each vaccine for the different age groups. Meningo, meningococcal; Pneumo, pneumococcal; Inf, influenza; HepA, hepatitis A; HepB, hepatitis B; Var, varicella; Zos, zoster.

Both perceived and actual knowledge were low. Less than 25% of physicians believed they knew most or all the adult vaccines. Figure 1 shows the vaccines physicians would recommend for different age groups. The most recommended vaccine was influenza. For all the vaccines except pneumococcal and zoster, the recommendation rate decreased as the age increased. When it came to Td/Tdap, only half of the participants would recommend it to any age group. As for adults aged 65 or older, 69% would recommend the influenza vaccine, only 57% would recommend the pneumococcal vaccine, and <20% would recommend the zoster vaccine. Sixty-one percent stated they knew the difference between the Td and Tdap vaccines but only 34% when it came to the zoster and varicella vaccines.

A knowledge score was calculated, and data analysis pursued as discussed in the methodology section. Both Shapiro–Wilk and the Q-Q plots indicated a lack of normality (Shapiro–Wilk *P*-value was <0.0005) and hence non-parametric methods were used to evaluate association between the knowledge determinants and the knowledge score. The maximum attained score was 25. The average score for all physicians was 9.5, with a standard deviation of 5.5. Table 3 presents the average knowledge score among the different demographic groups.

All demographic features were used as determinants of knowledge along with the number of patients seen each week and the perceived adult vaccination knowledge. Sex, age, citizenship, health authority, and the number of patients seen each week were not found to be associated with the knowledge score. Department, level of training, workplace, and perceived knowledge were fed into a multivariate linear regression model. A Breusch-Pagan test yielded a *P*-value of 0.091, indicating a lack of enough evidence for the presence of heteroskedasticity. The results of the model are shown in Table 4. All variables were significant except for workplace.

**Table 4:** Adult vaccination knowledge—multiple linear regression (ordinary least squares).

**Model terms**		**β-coefficient**	**RSE**	**t-Statistic**	***P*-value**	**2.5%**	**97.5%**
Intercept (β_0_)		4.347	0.606	7.178	**<0.0005**	3.156	5.538
Department **(P=0.0005)**	Others	–	–	–	–	–	–
	**Family Medicine**	**2.991**	**0.921**	**3.247**	**0.001**	**1.179**	**4.802**
	**Internal Medicine**	**1.593**	**0.691**	**2.305**	**0.022**	**0.234**	**2.951**
	Pediatrics	1.162	0.907	1.282	0.201	-0.621	2.945
Workplace **(P=0.006)**	Government Hospital	–	–	–	–	–	–
	Primary Healthcare	0.489	1.012	0.483	0.629	−1.501	2.479
	Private Clinic/ Hospital	1.089	0.727	1.499	0.135	-0.34	2.518
Training **(P=0.029)**	Intern House Officer	–	–	–	–	–	–
	**Resident**	**1.601**	**0.471**	**3.398**	**0.001**	**0.674**	**2.527**
	General Practitioner	0.042	0.623	0.068	0.946	-1.183	1.267
	Specialist	−0.007	0.649	−0.01	0.992	−1.283	1.269
	Senior Specialist	−0.41	1.047	−0.391	0.696	−2.469	1.649
	**Consultant**	**3.121**	**0.633**	**4.931**	**<0.0005**	**1.876**	**4.366**
**Perceived Knowledge (*****P*** ** < 0.0005)**		**1.227**	**0.283**	**4.329**	**<0.0005**	**0.669**	**1.784**
R-squared: 15.8%	Adjusted R-squared: 13.4%		**F**_**(10, 353)**_ **= 6.602; (*****P*** ** < 0.0005)**				

*Table shows the results of the Multivariate regression. Determinants of the knowledge score were fed into an Ordinary Least Squares Regression. Significant P-values are presented in bold*.

The model showed that family physicians and internists were more knowledgeable compared to other physicians (*P* = 0.001; *P* = 0.022, respectively), but pediatricians were not. However, there was no significant difference between internists and family physicians. Only residents and consultants showed more knowledge compared to medical interns (*P* = 0.001; *P* < 0.0005, respectively). Workplace showed no significant effect on the level of knowledge. Finally, better perceived knowledge is a significant predictor of better actual knowledge (*P* < 0.0005).

#### Knowledge Sources

**Figure 2:**
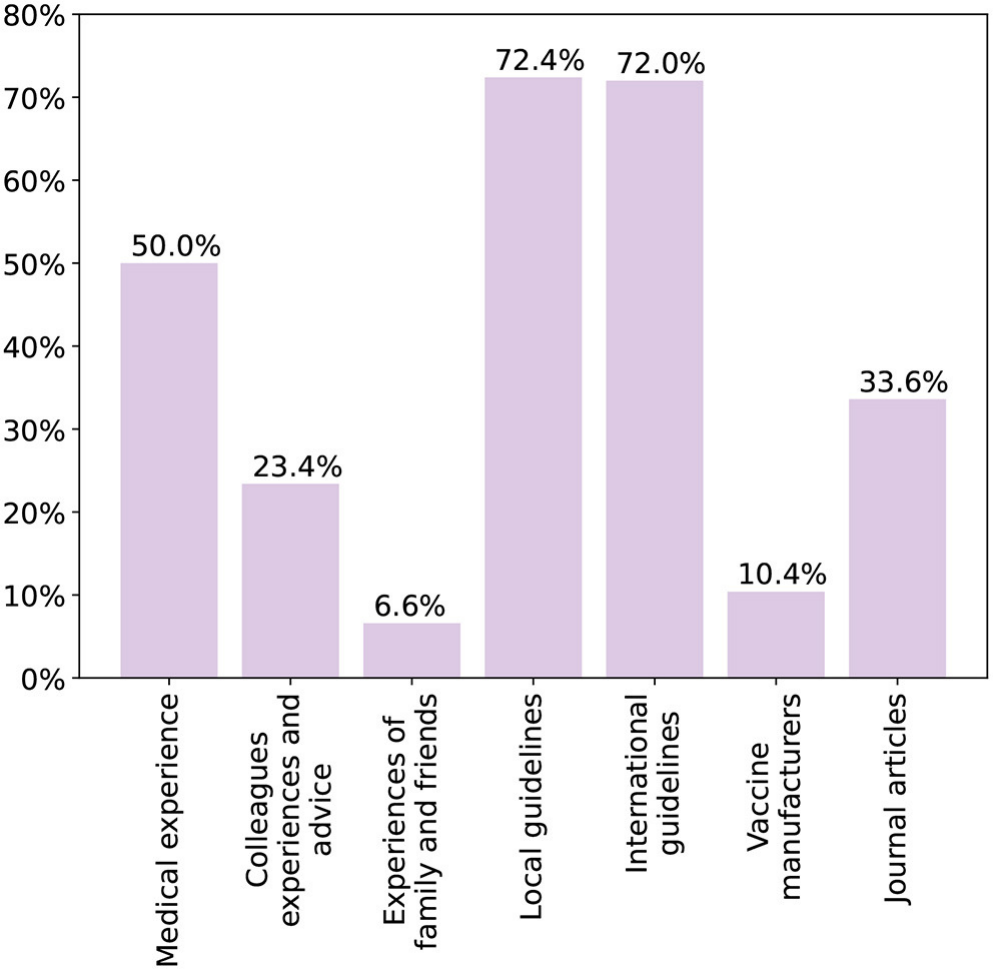
Main sources of knowledge of participating physicians.

**Figure 3:**
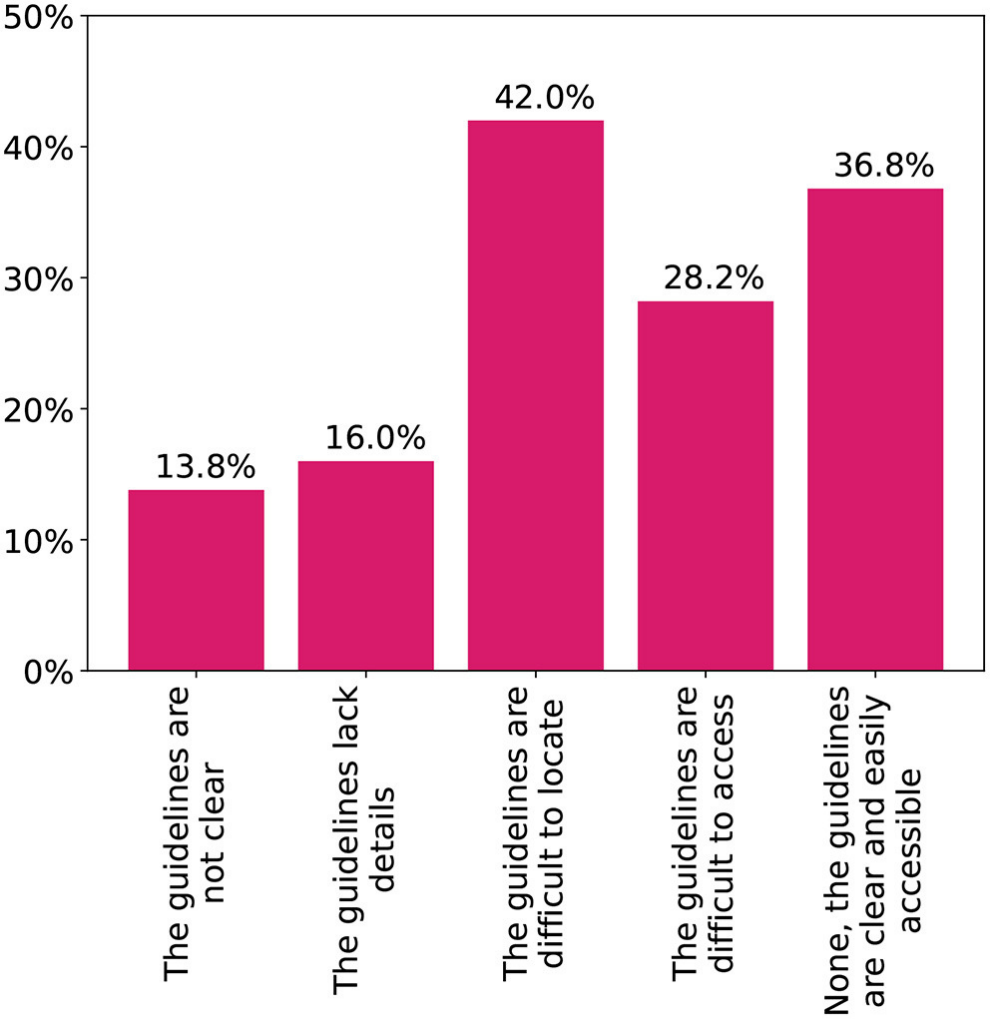
Difficulties participating physicians faced with the local adult vaccination guidelines.

Figure 2 shows the sources of knowledge on adult vaccines chosen by the physicians. Both international and local guidelines were the most common at 72%. Half of the participants also depended on their medical experience with the vaccines. Difficulties physicians faced with the local vaccination schedule are shown in Figure 3. Only 37% stated they faced no difficulties with finding or utilizing the guidelines. Several deficits were highlighted with 42% being unable to easily locate the guidelines and another 15% having issues with the clarity and content. Finally, 87% stated they would be interested in additional guidelines and training on adult vaccination.

## Discussion

### Adult Vaccination: The Global and Local Landscape

Adult vaccination is yet to achieve the same level of success that childhood vaccination has. Many organizations have looked at ways to improve adult vaccine uptake rates through a variety of different frameworks, actions, policies, recommendations, and programs. Yet, its rates remain dismally low locally, regionally, and even globally. In the USA, the rates of adult vaccine coverage in the community are low across the board with the highest uptake rate being for influenza at 44.8% (23). Data from the European Union presents an equally gloomy picture, with eight countries having elderly influenza vaccination rates of <50% and reaching as low as 4.3% (24). Moreover, out of 31 surveyed advanced economies, only 12 had a comprehensive adult vaccination schedule, with most recommendations geared toward older vaccines such as influenza and hepatitis B (25). As for the MENA region, data and research regarding adult vaccination is severely lacking. Influenza vaccination rates are dismally low in most countries, including the UAE and even among high-risk groups (26). Reasons for the low rates were plentiful, from negative media coverage and fear of side effects to low recommendations by physicians (7).

VPD monitoring and seroprevalence studies in the UAE are very scarce and most do not focus on adults. However, Abu Dhabi releases a quarterly communicable disease bulletin detailing the number of notified cases in the last three months; the latest of which (covering the second quarter of 2019) showed among adults aged 25 and above: 862 cases of chickenpox, 320 cases of hepatitis B, 1,756 cases of influenza, and an average of 25 cases for each of measles, mumps, and rubella (27). The yearly numbers can reach as high as ten times, given that this data represents the number of cases for a third of the population and only for a quarter of the year. Even more worryingly is the expected increased burden due to a rapidly aging population. In 2015, only 2.3% of the UAE's population was aged 60 years or older, compared to the West, where they represented more than a fifth of the population in most countries (4). However, the UAE's elderly population is expected to increase more than five-folds in 2030, reaching 11.3%, with some models predicting it as high as 14.3%. Hence, the effects of poor adult vaccine uptake rates have been masked by a young population, though not for long. As for vaccination studies, those are not extensive and deal with either specific vaccines or specific age groups; however, their results still show a lack of vaccine uptake and a high level of disease susceptibility (13–15).

The UAE's national agenda specifies a wide-ranging work program centered around six national priorities, the fourth of which is a “world-class healthcare” (28). Prevention would be an important pillar in such a healthcare system. Not only is this a fundamental component of a healthy good life, but it is also important as improved health leads to higher workforce participation and productivity. Hence, in any country, the goal is to integrate immunization into the healthcare system as a long-term sustainable service.

### Adult Vaccination and Physicians

In this study, the adult vaccination guidelines in the UAE were examined and compared to those in KSA and USA and the knowledge levels of UAE physicians were explored. The UAE guidelines for adult vaccination were found to be dated, unclear and not easily accessible. The guidelines additionally lacked extensive details regarding dosing, indications, and contraindications compared to the CDC's. The results also showed that physicians' knowledge of adult vaccines is lacking with most physicians being aware of this. Many were unable to recognize the vaccines recommended at every age group. Even the elderly vaccination schedule was not well-recognized, with a worryingly low number of physicians recommending the pneumococcal and influenza vaccines, the most fundamental for that age group. Future studies would have to establish whether the lower recommendation rates for the elderly stem from a lack of knowledge or a fear of side-effects in that vulnerable age group.

Very few studies have looked at adult vaccine knowledge in the region. However, AlMansoori et al. explored HPV vaccine knowledge in Al Ain city, UAE. Most knowledge questions were answered incorrectly by more than 40% of the participants and the HPV vaccination schedule was incorrectly recalled by more than 80% (29). In KSA, it was found that around three-quarters of physicians had poor knowledge regarding adult vaccines. The most cited reasons for the low adult vaccination rates were time constraints and a lack of up-to-date records (30).

#### Knowledge Sources

Healthcare professionals teaching and training programs usually assume that physicians would readily understand, support, and promote vaccination to the general population (31). Yet, it has been found that some HCPs access vaccine-questioning information online that can influence their confidence in vaccines (32). Social media platforms, which have been used to spread such information, can and have exacerbated vaccine confidence crises through negative campaigns, amplifying any existing anxieties (32).

In this study, most physicians reported depending on international and local guidelines with half also utilizing their medical experience. Similarly, a study among German Family Physicians found that 89.5% relied on STIKO, the German Standing Committee on Vaccination, as a primary source of information (33). However, in this study, most participants faced issues with the availability or clarity of the local guidelines. Confusion regarding the immunization schedule may lead to missed vaccination opportunities and reduce the effectiveness of the programs nationally. Moreover, nearly all participants were interested in additional training and guidelines for adult vaccination. Hence, the adult vaccine schedule should be reviewed and made easily accessible.

#### Promoting Vaccination

The importance of physicians in promoting adult vaccination cannot be understated. Patients have been consistently found to believe they do not qualify for vaccines, either due to not being in a high-risk group or having never been offered any vaccines (32). Physicians play an important role as both a source of information for patients and a promoter of vaccines. In the USA, the National Health Interview Survey found that vaccination coverage was higher among adults who had visited one or more physicians in the last year (23). When adult patients across Europe were questioned about their vaccination information sources, 65% reported physicians (31). Similarly, a physician's recommendation of a vaccine is vitally important, with multiple international studies linking it with better attitudes and practices toward adult vaccines (8, 12, 34, 35).

#### Barriers

There are deficiencies in the way doctors approach adult vaccination. Hurley et al. (9) found that doctors are prioritizing some vaccines over others and ranking vaccination below other preventative services. A quarter of American adults visited a physician that did not recommend the influenza vaccine, even though such a recommendation is associated with higher uptake (36). Locally, Rabei et al. (37) found that one out of every five parents in Al Ain community did not receive enough information regarding vaccination from physicians. Even more worryingly, Verger et al. (5) found that some physicians have stated a lack of confidence in the health authorities, expressed doubts regarding the safety of vaccines, or were not convinced of their utility. This highlights the rise of vaccine hesitancy among the front lines of medicine. Other major barriers of adult vaccination include an undervaluation of adult immunization (especially with misinformation perpetuated by anti-vaccination movements), and an inadequate infrastructure and cost system to support and improve access to adult vaccines (38). Finally, the importance of incorporating adult vaccination knowledge into medical school curricula cannot be understated, with future studies needed to evaluate the current status and any pitfalls.

#### Recommendations

Improving adult vaccination rates will require innovative solutions and a collective effort from both physicians and the community, most importantly, a shift from a passive immunization strategy to an active one. The local adult vaccination schedule needs to be updated and made more accessible to physicians. Physicians need to be informed regarding the availability and effectiveness of adult vaccines. Bach et al. found that provider education is an important step in promoting vaccines, with training being associated with higher vaccine delivery rates and increased vaccine championship (8). Additionally, there would need to be a unified plan across the country with clearly defined and publicly accepted vaccination goals. For this, there must be annual monitoring for adult vaccine uptake rates.

There should be increased patient engagement and education. Tan outlined some of the strategies to improve adult vaccination coverage which include (39):

1. Establishing the value of adult vaccines in the eyes of the public, policy makers, and healthcare professionals.
2. Improving access to recommended adult vaccines through stronger infrastructure and developing public-private partnerships to facilitate effective immunization behaviors.
3. Ensuring fair and appropriate compensation or subsidies for adult vaccines.

These strategies could focus on the patient-side or the provider-side, incorporating both the healthcare workers and regulatory bodies. A lot of work has been done to evaluate the effectiveness and utility of these techniques by global organizations such as the United States Community Preventive Services Task Force, the CDC's Quality Improvement Projects Targeting Immunization, and the National Vaccine Advisory Committee. Each aims to bring about improvements in adult vaccination rates through many new technologies like Reminder & Recall systems. While these associations focus on the American populace, their results may help outline how to improve adult vaccination locally. However, this again requires a local workforce responsible for implementing, measuring, and reporting on the effectiveness of these techniques locally.

Furthermore, the cost-effectiveness of adult vaccination is essential for it to thrive. A systematic review showed the majority of published studies reporting favorable cost-effectiveness profiles across the different age groups and medical conditions (40). However, given the different structure of the healthcare system in the UAE and demographic distribution, further studies would have to ensure these positive gains will hold for the country. Finally, the UAE's overall adult vaccination strategy also needs to be addressed. Any policy shifts can bring changes in the health system and improve health service delivery. Hence, future studies should aim to interview policy makers and outline the feasibility of such changes and any barriers.

### Validity

It is important to highlight the possible limitations of any research. This study depended on what physicians reported without any independent verification. Moreover, convenience and snowball sampling were used, yet care was taken to ensure responses were equally distributed among the three major health authorities in the country. No information was collected regarding the dosing that physicians would use. Additionally, as with all surveys, social desirability, and recall and response bias are possible. However, the survey being completely anonymous without any personal identifiers hints at the responses being authentic.

## Conclusions

Adult vaccination is a fledgling practice in the UAE that requires support, growth, and innovation. The burden of VPDs among adults is expected to increase. Physician's knowledge regarding adult vaccines is poor and the local guidelines are not clear or easily accessible. Physicians are highly receptive to more guidance and practice with adult vaccines. The local schedule needs to be updated and unified across the nation and physicians need be empowered and encouraged to promote adult vaccination. A local taskforce needs to be established to measure vaccine uptake rates, establish targets, and evaluate progress.

## Data Availability Statement

The raw data supporting the conclusions of this article will be made available by the authors, without undue reservation.

## Ethics Statement

The studies involving human participants were reviewed and approved by University of Sharjah-Research Ethics Committee. The patients/participants provided their written informed consent to participate in this study.

## Author Contributions

HB: conceptualization, methodology, validation, data curation, writing—original draft preparation, writing—review and editing, visualization, and supervision. KS: conceptualization, methodology, validation, data curation, software, formal analysis, writing—original draft preparation, writing—review and editing, and visualization. MH: conceptualization, methodology, validation, data curation, writing—original draft preparation, writing—review, and editing. FA: methodology, validation, data curation, writing—review, and editing. All authors have read and agreed to the published version of the manuscript.

## Conflict of Interest

The authors declare that the research was conducted in the absence of any commercial or financial relationships that could be construed as a potential conflict of interest.

## Publisher's Note

All claims expressed in this article are solely those of the authors and do not necessarily represent those of their affiliated organizations, or those of the publisher, the editors and the reviewers. Any product that may be evaluated in this article, or claim that may be made by its manufacturer, is not guaranteed or endorsed by the publisher.

## Abbreviations

CDC: centers for disease control and prevention
DHA: Dubai health authority
DOH/HAAD/SEHA: department of health
HCP: healthcare provider
KSA: kingdom of Saudi Arabia
MENA: middle east and North Africa
MOHAP: ministry of health and prevention
SCD: shared clinical decision
UAE: United Arab Emirates
USA: United States of America
VPD: vaccine preventable diseases
WHO: World Health Organization.
